# Fatty liver in uncomplicated type 2 DM is associated with impaired myocardial HEP metabolism, modulated by myocardial glucose uptake

**DOI:** 10.1186/1532-429X-11-S1-P93

**Published:** 2009-01-28

**Authors:** Jacqueline T Jonker, Rutger W van der Meer, Luuk J Rijzewijk, Adriaan A Lammertsma, Michaela Diamant, Johannes WA Smit, Albert de Roos, Johannes A Romijn, Hildo J Lamb

**Affiliations:** 1 Leiden University Medical Centre, Leiden, Netherlands; 2 Diabetes Centre, VU University Medical Centre, Amsterdam, Netherlands; 3 VU University Medical Centre, Amsterdam, Netherlands

**Keywords:** Coronary Artery Disease, Adenosine, Glucose Uptake, Fatty Liver, Diastolic Function

## Purpose

To study the associations between fatty liver (FL), insulin resistance, myocardial high-energy-phosphate (HEP) and glucose metabolism, and heart function in patients with uncomplicated type 2 diabetes mellitus (T2DM).

## Materials and methods

We studied 35 T2DM patients (Mean ± SD Hba1c = 7.0 ± 0.8%) without coronary artery disease or heart failure, as determined by echocardiography. ^1^H-MRS of the liver for the assessment of liver fat, myocardial ^31^P-MRS for assessment of myocardial HEP metabolism and MRI to determine left ventricular function were performed. Furthermore, a hyperinsulinemic, euglycemic clamp was performed to establish whole body insulin sensitivity. Moreover, PET with H_2_^15^O (fasting conditions) and [^18^F]-2-fluoro-2-deoxy-D-glucose (clamp conditions) were used to determine myocardial blood flow (MBF) and myocardial metabolic rate of glucose uptake (MMRglu) in a subgroup of 28 patients.

## Results

**Figure 1:**
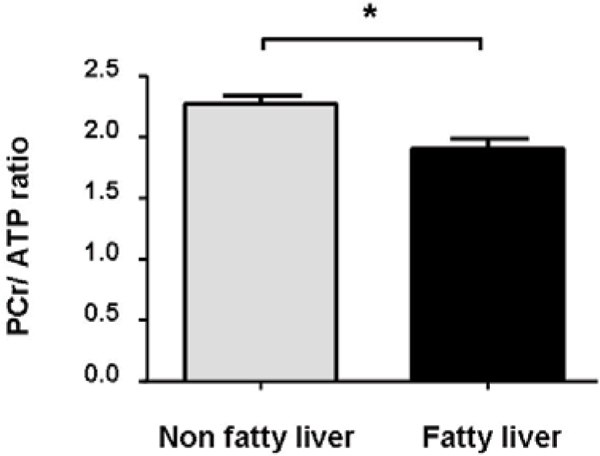
**Myocardial HEP metabolism in patients with DM2**. PCr/ATP ratio = ratio of phosphocreatine over adenosine triphosphate. *P < 0.05.

Patients with FL (liver: fat/water ratio>5%, n = 17) showed increased body mass index (29.5 ± 3.2 vs 27.2 ± 2.8 kg/m^2^, p < 0.05), reduced whole body insulin sensitivity (0.45 ± 0.48 vs 0.74 ± 0.44 (mg/kg·min)/(pmol/L), p < 0.05), and reduced MMRglu (0.21 ± 0.13 vs 0.34 ± 0.14 mmol/mL/min, p < 0.05), as compared with patients without FL, while MBF was not different. The ratio of phosphocreatine over adenosine triphosphate, a marker of myocardial HEP metabolism, was reduced in patients with FL (1.90 ± 0.35 versus 2.27 ± 0.29; p < 0.05), also after adjustment for BMI, and correlated to MMRglu (r = 0.43, p < 0.05). LV systolic and diastolic function were not statistically significantly different. Figure 1

## Conclusion

Fatty liver in patients with uncomplicated T2DM is associated with decreased myocardial HEP metabolism. In addition myocardial HEP metabolism is modulated by myocardial glucose uptake.

